# Effect of classic uvulopalatopharyngoplasty and laser-assisted uvulopalatopharyngoplasty on voice acoustics and speech nasalance

**DOI:** 10.4103/0256-4947.72266

**Published:** 2010

**Authors:** Mahmoud Y. Abu El-ella, Hatem E. Eldin, Khalid H. Malki, Mohamed M. Samir, Nasser H. Abd Al-Naser, Ahmed A. Mohamed

**Affiliations:** a From the Department of Otorhinolaryngology, Faculty of Medicine, Ain Shams University, Egypt; b From the Department of Otorhinolaryngology, Faculty of Medicine, South Valley University, Egypt; c From the Communication and Swallowing Disorders Unit and Research Chair of Voice and Swallowing Disorders, ENT Department, College of Medicine, King Saud University, Riyadh, Saudi Arabia

## Abstract

**BACKGROUND AND OBJECTIVE:**

Uvulopalatopharyngoplasty (UPPP) is a commonly used surgical technique for oropharyngeal reconstruction in patients with obstructive sleep apnea (OSA). This procedure can be done either through the classic or the laser-assisted uvulopalatopharyngoplasty (LAUP) technique. The purpose of this study was to evaluate the effect of classic UPPP and LAUP on acoustics of voice and speech nasalance, and to compare the effect of each operation on these two domains.

**PATIENTS AND METHODS:**

The study included 27 patients with a mean age of 46 years. All patients were diagnosed with OSA based on polysomnographic examination. Patients were divided into two groups according to the type of surgical procedure. Fifteen patients underwent classic UPPP, whereas 12 patients were subjected to LAUP. A full assessment was done for all patients preoperatively and postoperatively, including auditory perceptual assessment (APA) of voice and speech, objective assessment using acoustic voice analysis and nasometry.

**RESULTS:**

Auditory perceptual assessment of speech and voice, acoustic analysis of voice and nasometric analysis of speech did not show statistically significant differences between the preoperative and postoperative evaluations in either group (*P*>.05).

**CONCLUSION:**

The results of this study demonstrated that in patients with OSA, the surgical technique, whether classic UPPP or LAUP, does not have significant effects on the patients’ voice quality or their speech outcomes.

In patients with obstructive sleep apnea (OSA), uvulopalatopharyngoplasty (UPPP) is the most commonly used surgical procedure for enlarging the potential airspace in the oropharynx. The operation was first introduced by Fujita et al^1^ as a new technique to correct anatomic abnormalities in OSA patients. Laser-assisted uvulopalatopharyngoplasty (LAUP), later developed by Kamami,^2^ was aimed at progressively enlarging the oropharyngeal airspace by successive vaporizations of the uvula, soft palate, posterior tonsillar pillars and any redundant posterior pharyngeal mucosa. Postoperative pain, fatigue, hemorrhage, velopharyngeal insufficiency and nasopharyngeal stenosis were reported after both operations.

The phonatory characteristics after UPPP have been examined by different authors using objective (acoustical analysis) and subjective (perceptual evaluation) assessment techniques, but these studies mainly concentrated on the fundamental frequency, with contrasting results, and most of them investigated the effects of classical UPPP techniques.^3^ Whereas a number of authors reported significant voice changes after UPPP, other authors found no differences in voice and speech characteristics.^4^ There are only a few studies that have tested changes in voice and speech quality in patients with OSA after different palatal surgical techniques; therefore, uncertainty exists about speech and voice status in patients following UPPP and LAUP surgery. The aim of this study was to evaluate the effect of classic UPPP and LAUP on acoustics of voice and speech nasalance, and to compare the effect of each operation on these two domains to establish new concepts of management and a rationale for a therapeutic approach.

## PATIENTS AND METHODS

### 

The study was carried out at Ain Shams University Hospital, Cairo, Egypt. Twenty-seven adult patients (20 males and 7 females) with OSA who presented to the otolaryngology clinic between May 2007 and August 2008 were included in this study after having consented to participate. Their ages ranged from 18 to 57 years. Patients were selected when OSA was suspected based on history, clinical examination and polysomnography. According to the respiratory distress index (RDI), 7 patients had simple snoring without OSA (RDI, <5 events/hour), 5 patients had mild OSA (RDI, 5-25 events/hour), 7 patients had moderate OSA (RDI, 25-50 events/hour) and 8 patients had severe OSA (RDI, >50 events/hour). Patients subjected to previous operations for snoring and/or sleep apnea were excluded. In all subjects, OSA or severe snoring was established by polysomnographic examination. Each subject was assessed by an otorhinolaryngologist performing a complete ear, nose and throat examination to exclude voice disorders and nasal and ear diseases. For the nasopharyngeal and laryngeal examination, a fiberoptic nasopharyngolaryngoscope was used. The patients were randomly divided into two groups: 15 patients underwent classic UPPP, whereas 12 patients were subjected to LAUP. UPPP is a procedure designed to enlarge the potential airspace in the oropharynx. The uvula and part of the free edge of the soft palate were removed under general anesthesia. The procedure of LAUP operation was performed in the office using local anesthesia and a carbon dioxide (CO_2_) laser with special hand pieces to reduce and reshape the uvula and soft palate. Full-thickness vertical incisions were made on the edge of the soft palate approximately 1 to 1.5 cm in length on both sides of the uvula using CO_2_ laser at a power of 16 W in continuous mode. The resultant elongated uvula was then lasered off. The procedure progressively enlarges the oropharyngeal airspace by successive vaporizations of the uvula, soft palate, posterior tonsillar pillars and any redundant posterior pharyngeal mucosa. The following investigations were done for all the patients preoperatively and 6 months postoperatively.

#### Auditory perceptual assessment (APA)

Standardized speech samples were recorded using a high-fidelity tape recorder in a sound-treated room. The speech samples included a standardized passage, counting from 1 to 10 and sustaining the vowels/a/,/i/, and/u/. The recorded speech samples were perceptually judged for voice changes and nasality by three experienced phoniatricians. The degree of hypernasality was evaluated using a five-point scale (0=normal and 4=severe). Auditory perceptual assessment (APA) of voice was done according to the GRBAS (grade, roughness, breathiness, asthenia, strain) scale. Also, the parameters for overall grade of dysphonia, roughness and breathiness were rated on a five-point scale (0=normal and 4=severe).

#### Acoustic analysis of voice

A computerized speech lab (CSL model 4300 from Kay Elemetrics Corp., Lincoln Park, NJ, USA) was used for acoustic analysis of voice. The patient was seated in front of the microphone, and the mouth-microphone distance was kept constant at about 10 cm. The patient was asked to produce the sustained vowel/a/ in a flat tone at a comfortable pitch and at constant amplitude. Average pitch, jitter, shimmer and harmonic-to-noise ratio were measured for the two groups. Formant frequencies were done for the vowels/a/,/i/ and/u/. The first 2 formants (F1 and F2) were tested for these vowels. Acoustic analysis of voice was done preoperatively and 6 months postoperatively for all patients in both groups.

#### Acoustic analysis of speech (nasometric evaluation)

Using a nasometer (Model 6200 from Kay Elemetrics Corp., Lincoln Park, NJ, USA), the patient was instructed to produce a standardized oral sentence and a nasal sentence adapted to the Arabic language. Nasometric analysis of the patient’s speech was conducted to monitor possible postoperative nasality changes after operative procedures for each group.

The results were statistically evaluated by comparing the mean values of each preoperative and postoperative parameter for each operation, using the paired *t* test. The significance level was set to .05 throughout. The Statistical Package for Social Sciences, version 11 (SPSS Inc., Chicago, IL), was used for all statistical analyses.

### RESULTS

Twenty-seven patients participated in the study. Two patients were excluded, one because of an articulation disorder, and the other because of a common cold and nasal congestion on the day of testing. The selected group of subjects included 25 patients who completed the whole protocol of assessment preoperatively and postoperatively. The age of the study group ranged from 31 to 67 years (mean age, 46.6 years). There were 20 males and 7 females.

**Table 1** shows the APA results of the patients in both groups preoperatively and postoperatively. Differences in voice quality were not significant between the preoperative and postoperative evaluations for patients in each group (UPPP and LAUP). There was no statistically significant difference in either group between preoperative and postoperative assessments of acoustic quality. The *t* test showed no statistically significant change in average pitch, jitter, shimmer and harmonic-to-noise ratio before and after UPPP and LAUP for the sound/a/ (**Table 2**). Nasometric assessment did not show any statistically significant difference between the preoperative and postoperative nasalance scores in patients subjected to UPPP or those who underwent LAUP (**Table 3**). The formants F1 and F2 in each study group did not demonstrate any statistically significant changes between preoperative and postoperative assessments. The paired *t* test showed insignificant preoperative and postoperative differences in F1 and F2 values for/i/,/u/ and/a/ vowels in patients subjected to either UPPP (**Table 4**) or LAUP (**Table 5**).

**Table 1:** Comparison of preoperative and postoperative auditory perceptual assessments of voice.

	UPPP			LAUP		
Parameter	Preop	Postop	*P*	Preop	Postop	*P*
Overall grade	0.3 (3)	0.2 (3)	>.05	0.5 (0.4)	0.3 (0.3)	>.05
Roughness	1.2 (0.1)	1.5 (0.2)	>.05	0.91 (0.12)	1.2 (0.14)	>.05
Breathiness	0.4 (0.25)	0.51 (0.2)	>.05	0.61 (0.1)	0.53 (0.24)	>.05
Strain	0.8 (3)	0.6 (2)	>.05	1.59 (0.5)	1.3 (0.3)	>.05

Values are mean (standard deviation).

**Table 2:** Comparison of preoperative and postoperative values of acoustic parameters of voice.

	UPPP			LAUP		
Parameter	Preop	Postop	*P*	Preop	Postop	*P*
Average pitch (Hz)	167 (29)	165 (32)	<.05	170 (38)	168 (40)	<.05
Jitter (%)	0.21 (0.1)	0.19 (0.1)	<.05	0.13 (0.12)	0.13 (0.14)	<.05
Shimmer (%)	0.4 (0.25)	0.51 (0.2)	<.05	0.61 (0.1)	0.53 (0.25)	<.05
H/N (dB)	12.2 (3)	12.18 (3)	<.05	13.9 (3.5)	12.9 (4)	<.05

Values are mean (standard deviation).

**Table 3:** Comparison of preoperative and postoperative nasalance scores (%).

	UPPP			LAUP		
Parameter	Preop	Postop	*P*	Preop	Postop	*P*
Oral sentence	9.49 (1.19)	9.58 (1.03)	>.05	9.3 (1.5)	10.5 (3.1)	>.05
Nasal sentence	44.8 (4.3)	44.7 (3.5)	>.05	42.6 (4.48)	45.3 (9.0)	>.05

Values are mean (standard deviation).

**Table 4:** Comparison of preoperative and postoperative values of first formant (F1) and second formant (F2) of vowels /i/, /u/ and /a/ in patients subjected to UPPP.

	F1			F2		
Vowels	Preop	Postop	*P*	Preop	Postop	*P*
/i/	299 (22)	279 (77)	>.05	1808 (83)	1408 (78)	>.05
/u/	335 (30)	336 (27)	>.05	767 (46)	766 (43)	>.05
/a/	522 (143)	520 (140)	>.05	971 (39)	965 (48)	>.05

Values are mean (standard deviation).

**Table 5:** Comparison of preoperative and postoperative values of first formant (F1) and second formant (F2) of vowels /i/, /u/ and /a/ in patients subjected to LAUP.

	F1			F2		
Vowels	Preop	Postop	*P*	Preop	Postop	*P*
/i/	310 (21)	314 (20)	>.05	1841 (69)	1850 (58)	>.05
/u/	348 (20)	346 (21)	>.05	788 (18)	785 (17)	>.05
/a/	558 (30)	556 (32)	>.05	963 (34)	960 (33)	>.05

Values are mean (standard deviation).

## DISCUSSION

Patients and physicians are always worried about the effect of surgical modalities used for treatment of OSA on voice and speech. In a previous study performed by Mora et al,^5^ they found improvement in acoustic parameters and nasalance scores in all patients who underwent UPPP with the harmonic scalpel, 6 months after the operation, and they reported improvement in patients’ voice and speech after the operation. However, in our study, APA of the voice revealed no statistically significant voice changes postoperatively compared to the preoperative assessment. This was demonstrated in all patients who underwent either UPPP or LAUP. Our results also revealed that there were no statistically significant differences in acoustic voice parameters (pitch, jitter, shimmer and harmonic-to-noise ratio) among all patients before and after either UPPP or LAUP. The possible explanation for these findings is that acoustic voice parameters, including fundamental frequency and perturbation in frequency and loudness, reflect mainly the status of vocal folds’ vibration; however, the surgical technique, whether UPPP or LAUP, does not have a direct effect on the vocal folds or the larynx. This concurs with the findings by Van Lierde et al^6^ in their study of the voice and speech changes after UPPP. They found insignificant changes in the tested groups before and after the operation.

No statistically significant differences were found between preoperative and postoperative assessments in patients who underwent either UPPP or LAUP in nasalance of oral and nasal sentence. This means that the operative techniques did not affect the patients’ speech quality in terms of the nasal resonance. This concurs with the study done by Rihkanen and Soini,^7^ who mentioned that UPPP should not have a significant effect on speech characteristics as long as excessive nasality is not produced. On the other hand, Keilmann et al^8^ found that most of their patients had hypernasality and nasal escape of air 1 week after UPPP. Three to four months later, only one patient had hypernasality. Almost 1 year after surgery, velopharyngeal incompetence was no longer detected. This could be explained by the presence of functional hypernasality in the postoperative period due to the pain experienced postoperatively in the velum. However, later on the velopharyngeal valve worked competently in almost all patients.

The formants are features of the resonating cavities lying above the vocal folds. The formant is determined by the length and shape of the vocal tract, as these cavities are reshaped by changes in the positions of the tongue, jaw and lips. The first and second formants (F1 and F2) have frequencies for different vowels that generally reflect differences in tongue articulation. F1 frequency is related to the articulatory feature of tongue height. The vowels with a back tongue position have a low F2 frequency, whereas vowels with a front tongue position have a high F2 frequency. Thus F2 is generally related to the feature of tongue advancement (position of the tongue body on a back-front dimension).^9^

In the present study, on studying the preoperative and postoperative analysis of F1 and F2 values of different vowels (/i/,/u/ and/a/), we found no statistically significant differences between patients who underwent either UPPP or LAUP. This may be explained by the fact that F1 and F2 may be affected by the tongue shape and position and are not related to the shape of the soft palate.

The sample size of this study was relatively small, which is a potential limitation, but related studies have included almost similar number of patients.^5^,^6^,^10^ Those studies revealed results comparable to those of our study on the effect of UPPP on voice acoustics and speech nasalance.

In conclusion, classic UPPP and LAUP are two different surgical techniques used for oropharyngeal reconstruction in patients with OSA. The results of this study revealed that the surgical technique, whether classic UPPP or LAUP, does not have any significant effects, either on the patients’ voice quality or on the nasal resonance of their speech.
